# An effective detection model based on YOLO for pore defects in additive manufacturing

**DOI:** 10.1038/s41598-026-43970-2

**Published:** 2026-03-21

**Authors:** Rui Ni, Siwen Xu, Hanning Chen, Maowei He, Zhaodi Ge, Xiaodan Liang

**Affiliations:** 1 School of Mechanical Engineering, Tiangong University, Tianjin, 300387 China; 2 School of Computer Science and Technology, Tiangong University, Tianjin, 300387 China; 3 School of Control Science and Engineering, Tiangong University, Tianjin, 300387 China; 4 Shaoxing Keqiao Institute of Tiangong University, Shaoxing, 312030 China

**Keywords:** Selective laser melting, YOLO, Image segmentation, Pore defect, Chebyshev polynomial, Engineering, Mathematics and computing

## Abstract

Microscopic imaging serves as a crucial method for assessing the quality of selective laser melting (SLM). Traditional approaches rely on manual inspection, which limits their efficiency and reproducibility. To address the demand for defect detection and analysis, this paper proposes a synergistic method for analyzing pore defects in microscopic images, integrating image segmentation with polynomial fitting. We designed a high-performance image segmentation model. Its capabilities are enhanced through an adaptive curved learning rate adjustment strategy, an attention-based feature extraction module, and a lightweight feature fusion network. Additionally, the model automatically calculates and quantifies the pixel proportion of pore defects within micrographs. Experiments conducted on a constructed SLM pore defect microscopic image dataset demonstrated excellent performance, enabling effective calibration and quantification of defect information. Chebyshev polynomials are employed to fit the nonlinear relationship between key process parameters and porosity. Based on these results, we conducted an in-depth analysis of how different process parameters influence pore defect formation, revealing the intrinsic correlation between process parameters and defects. This study provides an effective automated detection and analysis tool for SLM quality assessment and analysis.

## Introduction

Additive manufacturing (AM) constitutes an integrated manufacturing technology that fabricates three-dimensional parts through sequential layer-by-layer material deposition, enabling creation from scratch^1^. Compared to traditional manufacturing techniques, AM demonstrates significant advantages in design flexibility, material utilization, and the formation of complex structures. Selective Laser Melting (SLM)^2^, as a significant AM technology, demonstrates outstanding performance in processing, including elevated strength, outstanding toughness, and excellent corrosion resistance^3^. It is widely utilized across strategic industries such as aerospace^4^, automotive manufacturing^5^, and medical devices^6^, becoming a vital force for modern manufacturing.

For SLM fabricated parts, the intense interaction between the laser beam and metal powder induces various microstructural defects, which markedly influence the manufacturing quality and mechanical properties of the components^7^. Pore^8^, a primary microstructural defect, includes keyhole porosities induced by excessive laser energy input^9^, lack-of-fusion (LoF) porosities resulting from insufficient energy density^10^, spatter-related LoF defects^11^, and gas entrapment porosities originating from incomplete gas escape during melt pool solidification^12^. A large number of irregular porosities can induce stress concentration, which may initiate macroscopic imperfections including cracks and fractures^13^. Therefore, effective control of porosity emerges as a critical prerequisite for improving the quality and service performance of SLM-fabricated components.

Common microstructure observation imaging techniques include X-ray imaging, scanning electron microscopy (SEM), computed tomography (CT), ultrasonic testing, and laser diffraction^14^. These techniques play a crucial role in characterizing microstructural defects but require extensive post-processing and data computation. Sun et al. employ SEM to characterize the microstructure of soft magnetic material samples fabricated by direct energy deposition (DED)^15^. Peng et al. employed multiple diffraction techniques to measure anisotropy in LPBF-fabricated 316L components, mitigating the influence of macroscopic defects through evaluation and statistical analysis^16^. Ultrasonic testing can be employed as a reliable non-destructive method for assessing defect characteristics in parts fabricated by Fused Filament Fabrication (FFF)^17^. In the literature^18^, X-ray computed tomography (XCT) is employed to inspect defects in AM components, it highlights the inherent limitations, particularly the necessity for superior scan quality evaluation criteria beyond voxel size. XCT has also been utilized for surface characterization of polymer AM parts, as demonstrated in^19^, where it is employed to assess surface roughness for enhanced process quality. Notably, XCT has been extensively utilized for non-destructive porosity analysis in metallic additive manufacturing (AM) components^20^. Manufacturing defects significantly influence the quality and performance of industrial products. Consequently, developing a novel defect identification method based on micrographs is essential for enhancing detection speed and establishing the constitutive relationship between defects and processing parameters, thereby guiding high-quality production.

In recent years, object detection technology has seen increasingly widespread application in industrial inspection^21^. Image segmentation, as a critical image processing technology, enables researchers to rapidly visualize the microstructural features of fabricated components. Deep learning^22^, as a pivotal technique in object detection, has garnered significant attention for its exceptional performance in detecting specific objects by analyzing feature relationships between pixels within the entire image. A U-Net-based convolutional neural network (CNN)^23^ is employed for defect segmentation in AM components, demonstrating strong potential for industrial quality control applications. In^24^, deep learning-based instance segmentation is employed to detect defects in 18Ni300 samples fabricated by LPBF, followed by a quantitative analysis correlating process parameters with mechanical properties. Surovi et al. utilized acoustic signals to develop machine learning models for identifying geometric defects during Wire Arc AM, demonstrating accurate detection of defective segments in Inconel 718^25^. Acharya et al. developed a deep learning-based defect detection method for XCT images of AM parts^26^, demonstrating enhanced capability in identifying defects. Tang et al.^27^ utilized the simulated XCT data combined with a 3D-CNN model to develop a method for predicting porosity in AM components. Although deep learning demonstrates significant potential in defect detection, conventional CNN models entail prohibitively high computational costs, thus highlighting the urgent need for more efficient and lightweight deep learning models.

YOLO is a single-stage object detection model that has gained significant attention in manufacturing applications due to its lightweight architecture and rapid inference speed. Su et al. proposed a virtual polarization filtering algorithm to enhance image quality prior to YOLO-based defect detection under complex lighting conditions^28^. In^29^, YOLO was employed to detect pores from XCT-derived images. Huang et al.^30^ adapted a modified YOLOv8 model for surface defect identification in wire arc additive manufacturing (WAAM) processes. Sani et al.^31^ implemented defect detection for fused deposition modeling (FDM) to minimize material waste, while Wen et al.^32^ quantified metal AM CT data using an improved YOLOv7 framework and successfully predicted structural yield stress. These approaches provide effective tools for defect detection; however, current research primarily focuses on defect identification and segmentation, with limited progress in quantitative correlation analysis of defect characteristics.

This work presents an efficient and accurate image instance segmentation method to accelerate the analysis of micrographs for defects in traditional AM, reduce detection costs. This method has been effectively applied to SLM-manufactured AlSi10Mg components, accurately segmenting pore defects in the surface micrographs of the samples. Based on the pixel data from the segmentation results, the proportion of these defects on the sample surface is calculated, aiding in the discovery of constitutive relationships between pore defects and process parameters.

In this paper, Section “Methodology” discusses the sample preparation methods and dataset construction, followed by an introduction to the proposed semantic image segmentation model and the method for establishing mapping relationships between pore defects and process parameters. Section “Result and discussion” presents experimental validation of the proposed model’s performance and discusses how processing parameters influence pore formation based on empirical analysis. Conclusively, Section “Conclusion” consolidates the main methodological innovations and findings of this research.

## Methodology

In this work, a synergetic analytical method that combines instance segmentation with polynomial fitting is proposed for the analysis of defect formation in AM. Using AlSi10Mg metal powder, 49 SLM specimens under varying process parameters are fabricated, and their high-resolution surface micrographs are experimentally obtained. An instance segmentation dataset is established manually, comprising high-resolution microstructural imagery paired with labeled defect annotations. Subsequently, a YOLO-based instance segmentation framework is proposed specifically for pore defect detection in micrograph images, and its effectiveness is validated to utilize a constructed defect instance segmentation dataset. Finally, mapping relationships between pore defects and process parameters are established through pixel data from image analysis and mathematical methods.

### Materials

#### Sample preparation

AlSi10Mg metal powder is selected as the base material for fabricating the test specimens. The LiM-X260A experimental apparatus is used to produce cubic samples measuring 10 mm × 10 mm × 10 mm under protective atmosphere. During printing process, the experimental specimens employed an interlayer rotation angle of 67° and a layer thickness of 0.03 mm. To obtain specimens with varying properties, the laser scanning speeds ranged from 700 to 1900 mm/s, while the laser power varied between 160 and 400 W, achieving an energy density range of 20.1 to 136.1 J/mm^3^.

#### Imaging and characterization

Surface characteristics of the specimens are characterized by optical microscope (OM). All specimens are ground with sandpaper ranging from 180 to 1200 grit, followed by polishing with SiO₂ medium polishing compound. Subsequently, the metallographic surface of the specimens is observed under OM at 20 magnifications. Starting from one corner of the specimen, it is moved incrementally in stages to capture images of the entire observed surface. Ultimately, a total of 980 OM images is obtained from the specimen.

### Model design

You Only Look Once (YOLO), introduced by Redmon et al., is a representative single-stage object detection framework widely used in industrial image tasks^33^. This work employs YOLOv5^34^ for instance segmentation of pore defects. Researchers in recent years have mostly conducted further studies on the relatively stable foundational models of YOLOv3, YOLOv4, and YOLOv5, with YOLOv5 being more mature and refined^35^.

In this study, the proposed model comprises three core architectures: Backbone, Neck, and Head. Backbone performs feature extraction from input images. Multi-scale features extract at different stages undergo information fusion through the Neck. After the fused information enters Head at the model’s terminal end, it is distributed to three detection heads with default resolutions of 20 × 20, 40 × 40, and 80 × 80, where non-maximum suppression (NMS) is utilized to compute the target regions.

**Fig. 1:**
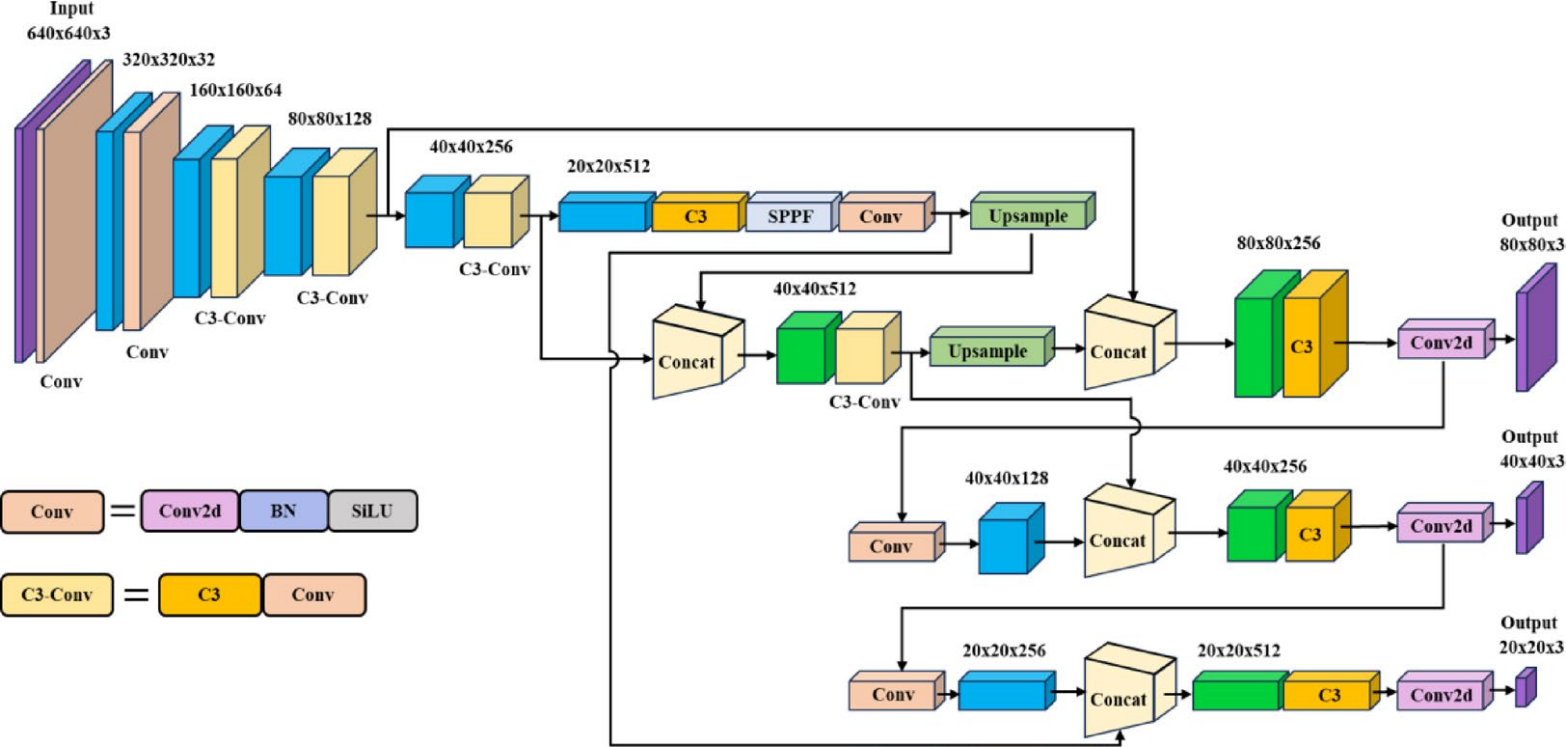
The improved structure framework.

We extended the functionality of YOLOv5, with the improved model illustrated in Fig. 1. The following modifications are introduced to enhance detection performance:

- An adaptive curved learning rate adjustment strategy is proposed to improve the model’s adaptability to diverse datasets.
- A novel attention-driven feature extraction module is developed to strengthen the backbone network’s capacity in extracting features.
- A lightweight feature fusion network is developed to improve fusion effectiveness while minimizing model complexity and size, balancing performance and applicability.

#### Adaptive curved learning rate adjustment strategy

The baseline model employs a linear decay learning rate adjustment strategy, which provides consistent attention throughout the entire training process. However, we believe that adjusting the learning rate curve on account of the dataset’s characteristics and distribution can improve the model’s training speed and performance. For datasets with distinct features and uniform distribution, a linear learning rate is appropriate. However, for datasets where some categories exhibit ambiguous features or complex characteristics, the model is prone to getting stuck in local optima during training, leading to reduced learning speed and performance. To mitigate this, we propose an adaptive curved learning rate adjustment strategy that maintains a higher rate in early stages to facilitate broader exploration of the parameter space, thus avoiding premature convergence. The formulation of the proposed strategy is as follows:

1 $$lr = lr0 * \left( {lr_{min} + \left( {lr_{max} - lr_{min} } \right) \times \left( {1 - \left( {\frac{x}{{N_{e} }}} \right)^{a} } \right)^{b} } \right)$$

In Eq. (1), *lr*_0_ represents the initial learning rate. *lr*_*max*_ and *lr*_*min*_ denote the maximum and minimum ratios for learning rate decay strategy, respectively. *x* signifies the current epoch and *Ne* serves as the max epoch. The proposed strategy can control the rate of learning rate decay at different stages of training by adjusting parameters *a* and *b*, thereby enhancing the model’s adaptability to diverse datasets. In this work, *a* and *b* are set to 2.2 and 1.6.

#### Attention-based feature extraction module

To address the insufficient attention to detail in the feature extraction process of the original backbone, an attention-based feature extraction module C3CA is constructed by integrating a spatial channel attention mechanism with C3 module. This mechanism utilizes the importance evaluation of information, assigns different weights to each channel, and enhances the network’s ability to capture the critical information across different channels and spatial locations.

The Convolutional Block Attention Module (CBAM) is an efficient, lightweight spatial-channel attention module that simultaneously consists of both channel attention and spatial attention components. Its computation is summarized in Eq. (1) and (2):

2 $$F^{\prime} = F \otimes M_{C} \left( F \right)$$

3 $$F^{\prime\prime} = F^{\prime} \otimes M_{S} \left( {F^{\prime}} \right)$$

where, *F* is input images. *M*_*S*_ denotes spatial attention.* M*_*C*_ represents channel attention.

**Fig. 2:**
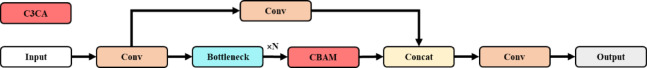
The structure of C3CA.

The CBAM attention module comprises two steps. First, each channel of the input image undergoes max and average pooling separately, yielding two feature maps of *C* × 1 × 1 dimension. Features are extracted using a Multilayer Perceptron (MLP) channel convolution module with a specific compression and restoration ratio. Two channel attention maps are added together to activate them, followed by multiplication and fusion with the input feature map to generate the channel attention feature map. Second, the resulting feature map undergoes spatial max and average pooling to form two 1 × *H* × *W* maps. Concat and compute two feature maps to obtain the global spatial attention map. Multiply and fuse the global spatial attention map with the channel attention feature maps to obtain the output feature map. The attention mechanism dynamically calibrates weights in channel and spatial dimensions, enabling the model to focus on critical morphological features of defects while suppressing interference from non-defect features during training. Figure 2 shows C3CA module, which replaces C3 module in Backbone to enhance feature extraction capabilities and improve model accuracy and generalization.

#### Lightweight feature fusion network

**Fig. 3:**
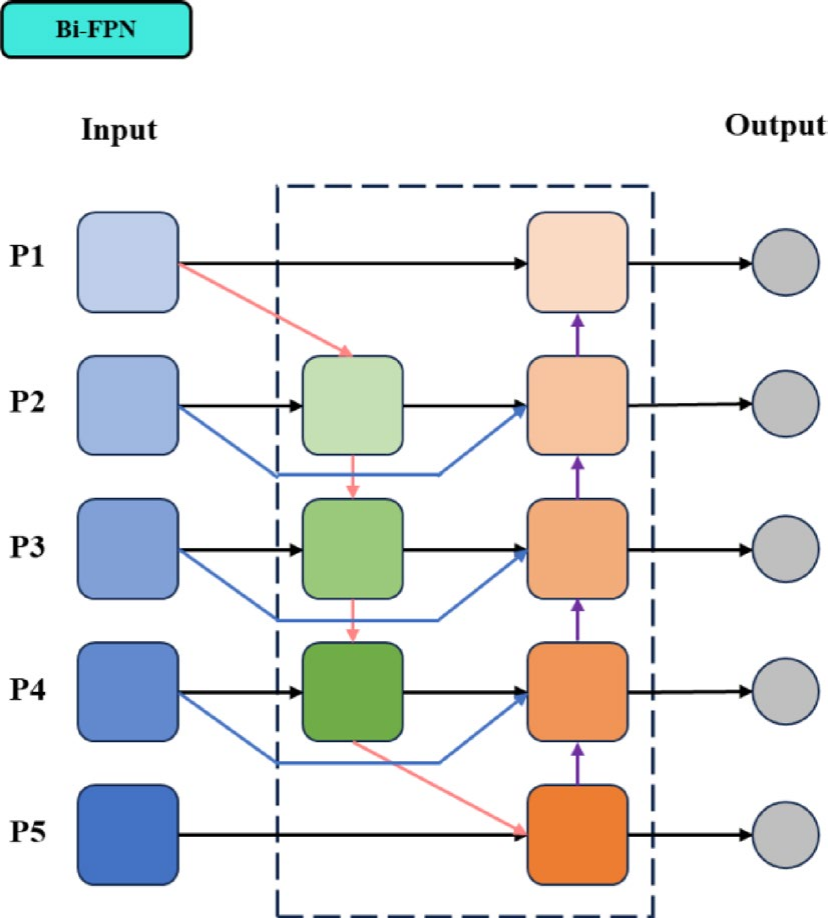
The structure of Bi-FPN.

The Neck consists of FPN and PAN for multi-scale feature fusion. However, this network architecture exhibits diminished feature extraction capabilities when confronted with datasets exhibiting complex characteristics. This work employs the Bidirectional Feature Pyramid Network (Bi-FPN)^36^ based on EfficientNet to enhance the fusion process. While most feature fusion methods treat multi-scale features equally, Bi-FPN introduces weight coefficients that assign weights to features based on their contribution to the fusion, enabling faster and more effective multi-scale feature integration. This network architecture shows as Fig. 3.

**Fig. 4:**
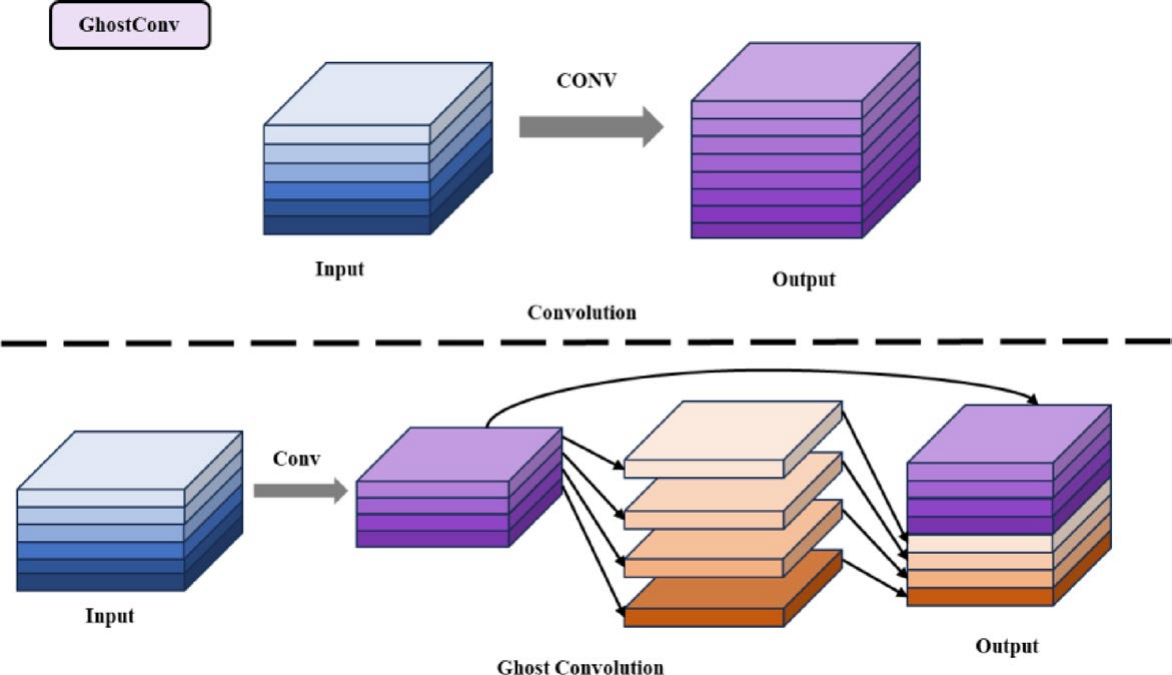
The structure of GhostConv.

To reduce model size and complexity, a lightweight convolution module called the GhostConv module is employed in Neck. Traditional convolutions produce numerous similar feature copies in the output. The GhostConv module obtains feature copies (ghost feature maps) through a series of linear transformations to achieve computational lightness. Figure 4 compares the GhostConv module with the traditional convolution module. The Lightweight Feature Fusion Network structurally compresses parameter size while enhancing feature representation efficiency and utilization. This architectural design mitigates overfitting tendencies and strengthens structural simplicity and robustness.

### Defect quantification

Leveraging a pre-trained image instance segmentation model, an automated data calculation method is established to automatically compute the proportion of pore defect pixels relative to the total pixel count using the segmentation results’ pixel data. Since the surface micrograph data consists of continuous, progressively captured images of identical dimensions, the quantitative instance segmentation defect value for the porosity of each specimen’s measured surface represents the average of its corresponding images.

### Process parameter fitting analysis

Based on the porosity data obtained from the proposed model, a Chebyshev polynomial is employed to establish a mapping relationship model between laser power, scanning speed, and porosity. Owing to its superior approximation properties over intervals, the Chebyshev polynomial effectively describes the complex nonlinear relationships in SLM processes. After normalizing the laser power and scanning speed, a high-order binary Chebyshev polynomial model is established. The model’s undetermined fitting coefficients are solved using regularized least squares, and the coefficient R2 is utilized to evaluate model accuracy and facilitate iterative coefficient refinement.

## Result and discussion

### Model parameter and evaluation metric

Both the developed model and the baseline architecture are constructed and deployed utilizing PyTorch. All models are trained for the same epochs, with performance metrics saved after each epoch. Following training, all models are assessed using identical evaluation metrics. Specific model parameters and experimental settings are detailed in Tables 1 and 2:

**Table 1:** Parameters setting.

Parameter	Value
Learning rate	0.01
Momentum	0.937
Batch size	4
Epochs	300

**Table 2:** Training equipment configuration.

CPU	GPU	OS	RAM	VRAM
Intel i9-12900H	Nvidia RTX 3060	Windows 11	16 GB	6 GB

*Mask mAP* A model accuracy evaluation metric, it serves as the core evaluation indicator for instance segmentation tasks, measuring the model’s accuracy in pixel-level segmentation.

*FLOPS* Floating-point computation rate per second, it serves as a key indicator for evaluating model complexity. As the FLOPS of a model decreases, both its computational demands and hardware requirements diminish.

*Model Size* Measured in megabytes, it serves as a key criterion for evaluating model complexity. As model size decreases, the number of parameters and storage requirements diminish, enhancing the model’s adaptability to a wider range of hardware devices.

### Dataset construction

In this work, all specimens are examined under optical microscope to identify pore defects and capture corresponding images, establishing the SLM AlSi10Mg Pore (SLMAP) dataset explore the interdependence between pore defects and process parameter. All microscopic images underwent manual annotation of pore defect features using labelImg software. The dataset comprises 980 images of 4000 × 3000 pixels with corresponding annotations, with 20 OM images captured per specimen. The micrograph pixel resolution is calculated to be 0.7 μm/pixel by shooting a 1 mm standard ruler, with a field of view (FOV) of 2.8 mm × 2.1 mm. Step size is synchronized with FOV dimensions, ensuring overlap rates below 5% through real-time preview during image acquisition. All images are captured using the built-in illumination system of the same optical microscope, comprising a single 5 W warm color LED source. All images are confirmed to be in focal clarity through real-time preview during the acquisition process, eliminating samples with abnormal exposure. The acquired micrographs undergo independent reviewed by two researchers, selecting samples with clear and distinguishable pores and matrix, and no extraneous contaminants as dataset entries. For training convenience, image resolution is adjusted to 768 × 512 during model training. For model validation, the dataset undergoes random partitioning into training and testing subsets with an 8:2 ratio.

### Model ablation experiment

Due to the multiple improvements of the proposed model over the baseline model, it is essential to investigate the impact of each enhancement on the model’s accuracy and computational complexity. The SLMAP dataset is used as input to train models for each model variant incorporating different improvements, with parameter settings remaining consistent with Section “Model parameter and evaluation metric”. Owing to constraints on training time and computational resources, training each model for 50 epochs is sufficient to demonstrate the effectiveness of the improvements. Table 3 lists the evaluation metrics: Mask mAP 50, Mask mAP 50:95, FLOPS, and model size.

**Table 3:** Comparison of the accuracy results of different improvements.

C3CA	Bi-FPN	Lightweight	Mask mAP50 (%)	Mask mAP50:95 (%)	Size (MB)	FLOPs
–	–	–	77.9	38.8	19.8	37.8G
√	–	–	80.8	41.2	21.5	41.6G
–	√	–	80.1	39.8	19.8	37.8G
–	–	√	80.8	41.0	17.5	35.9G
√	√	–	81.6	41.6	21.5	41.6G
√	–	√	81.5	42.0	19.2	39.7G
–	√	√	81.8	41.6	17.5	35.9G
√	√	√	83.5	42.6	19.2	39.7G

**Fig. 5:**
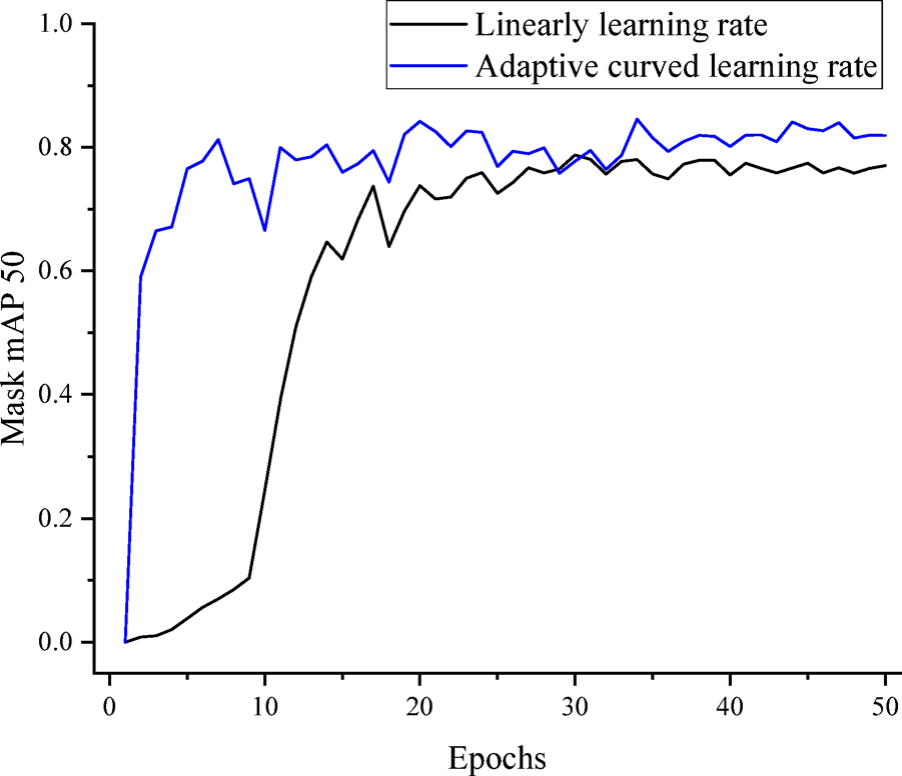
Mask Map50 curve of the proposed strategy and linear learning rate.

In SLM manufacturing, the morphology of pore is influenced by multiple factors including material quality, process parameters, and processing environment, exhibiting a degree of irregularity. Consequently, the boundaries of instances in SLMAP dataset demonstrate significant complexity. As shown in Fig. 5, the training curve demonstrates that the proposed adaptive curved learning rate adjustment strategy significantly improves detection accuracy. This result validates that the strategy establishes a correlation between training epoch and convergence, thereby providing a more appropriate learning intensity during the training phase to achieve superior performance.

In SLM micrographs, the matrix of the fabricated part, which can vary in quality, often acts as a complex background that adversely impacts the feature extraction of pore defects. Based on the experimental outcomes detailed in Table 3, the findings demonstrate that incorporating the attention-based feature extraction module significantly enhances model performance, enabling more effective extraction of pore defect features. Mask mAP 50 and Mask mAP 50:95 improved 2.9 and 2.4, respectively. From the perspective of multi-scale feature fusion, the adoption of the new feature fusion network approach resulted in improvements of 2.2 and 1.0 for Mask mAP 50 and Mask mAP 50:95, respectively. Concurrently, the integration of the lightweighting module reduces model size and FLOPS by 2.3MB and 1.9G, respectively. This confirms that the lightweight module effectively lowers the model’s demands on computational resources and device configuration. Overall, this work achieves a superior model in terms of performance.

### Pore identification and quantitative calculation

To better evaluate the performance of our proposed model, comparative evaluations are conducted against other models trained using the SLMAP dataset. Table 4 compares the training results of all models after 300 epochs, demonstrating that the proposed model achieves superior overall performance relative to all models.

**Table 4:** Comprehensive comparison of the proposed model versus alternative models trained on SLMAP dataset.

Model	mAP50	Precision	Recall	Size	Parameters	FLOPs
YOLOv5s	82.3	84.0	78.1	19.9MB	9,765,939	**37.8G**
YOLOv6s	82.9	83.6	77.4	33.8MB	16,748,883	56.7G
YOLOv8s	83.1	**85.2**	77.7	23.9MB	11,779,987	42.4G
YOLOv10s	83.6	83.7	77.8	**18.8MB**	**9,170,323**	40.5G
Proposed model	**85.6**	**85.2**	**80.0**	19.2MB	9,415,767	39.7G

The best results are highlighted in bold.

In Table 4, YOLOv6s prioritizes improvements in training efficiency and multi-platform adaptability, resulting in slightly suboptimal performance on SLMAP dataset. YOLOv8s enhances feature extraction capabilities through cross-stage partial connections to achieve superior performance. YOLOv10s employs a dual-label assignment strategy to address the issue of redundant prediction bounding boxes, thereby offering advantages in terms of model size and parameter count. Compared to YOLOv5s, YOLOv6s, and YOLOv8s, the proposed model achieves mAP50 improvements of 3.3, 2.7, and 2.5, respectively, while reducing model size by 0.7, 14.6, and 4.7, respectively. Due to its simplest computational process for network architecture and modules, YOLOv5s requires the lowest floating-point operations, resulting in the smallest FLOPs. Although the proposed model has slightly larger model size and parameter count than YOLOv10s, it achieves a 2.0 enhancement in the accuracy metric mAP50. In summary, the combined effect of multiple optimization mechanisms enhances detection accuracy, while the application of lightweight modules improves computational efficiency.

**Fig. 6:**
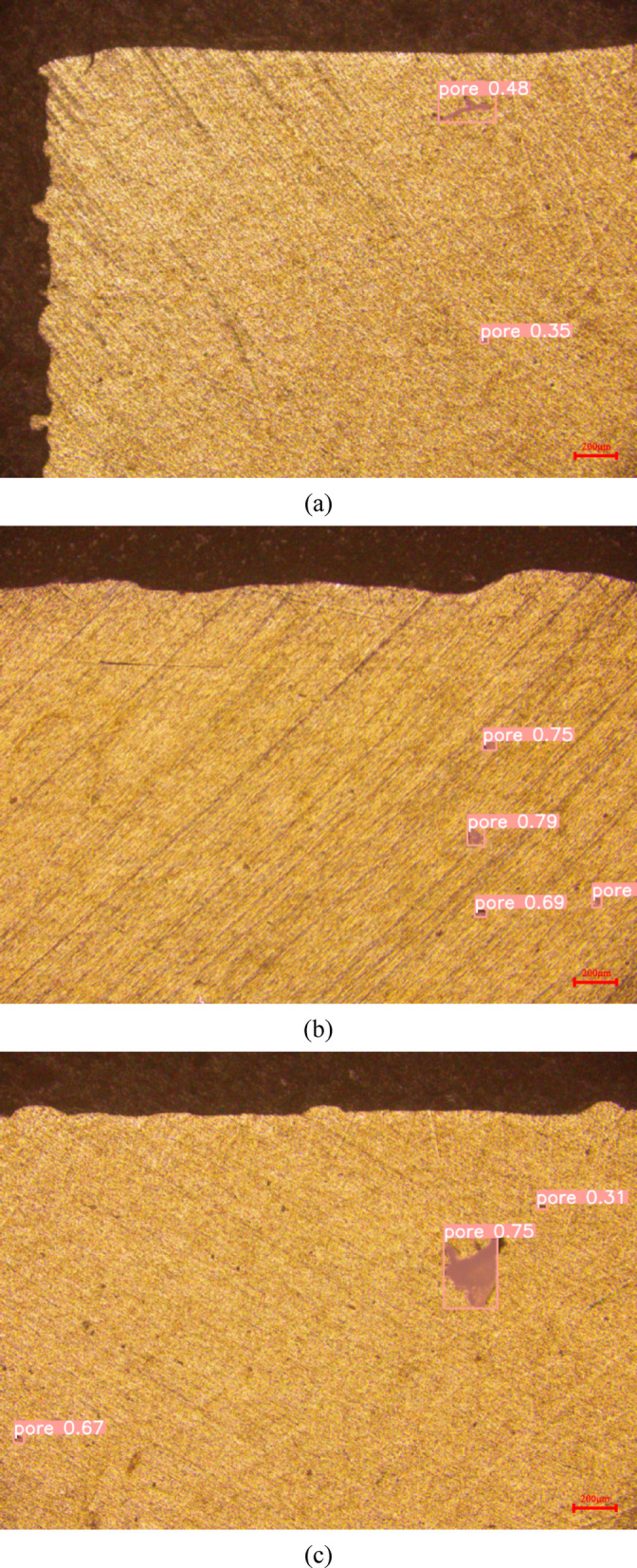
Example segmentation results (**a**), (**b**), and (**c**) on SLMAP dataset.

Figure 6 shows the segmentation results for a part of the dataset images. As can be seen from the figure, the segmentation effectively covers the regions where pore defects are present and accurately delineates the boundaries of these defects. Although minor segmentation discrepancies arise in regions with weak gradient features of irregularly shaped pores, as exemplified by the defect with 0.75 probability in Fig. 6c, the proposed model maintains strong overall segmentation performance. These results demonstrate that the current model can accurately segment and identify pore defects of varying morphologies and sizes.

**Fig. 7:**
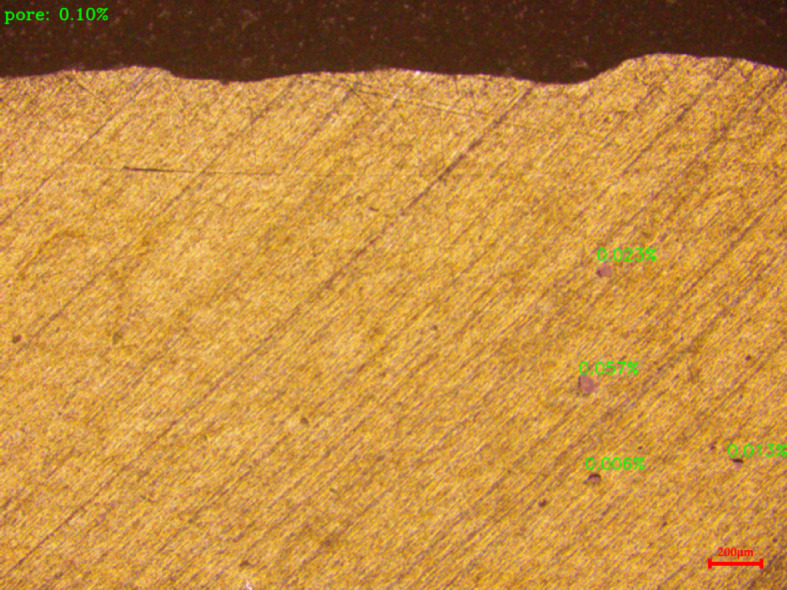
Example results of the pixel proportion calculation.

Based on reliable segmentation results, the proposed model achieves automatic calculation of the pixel proportion of pore defects by extending the script functionality. Figure 7 displays partial results of the pixel proportion calculation. Analysis indicates that the error between the model’s automatically calculated pore pixel proportion and manual measurements is controlled within 5%, validating its reliability as a quantitative analysis tool. This method effectively enhances the statistical processing efficiency of pore defect information in microscopic images.

### Influence of process parameters on pore defects

**Fig. 8:**
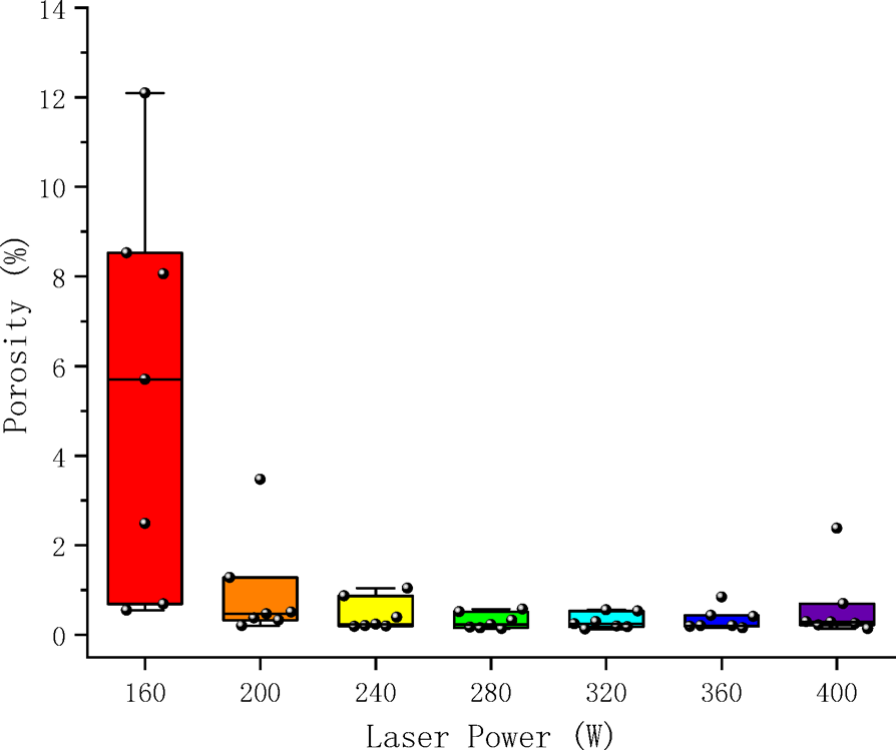
Scatter box plot of porosity and laser power.

**Fig. 9:**
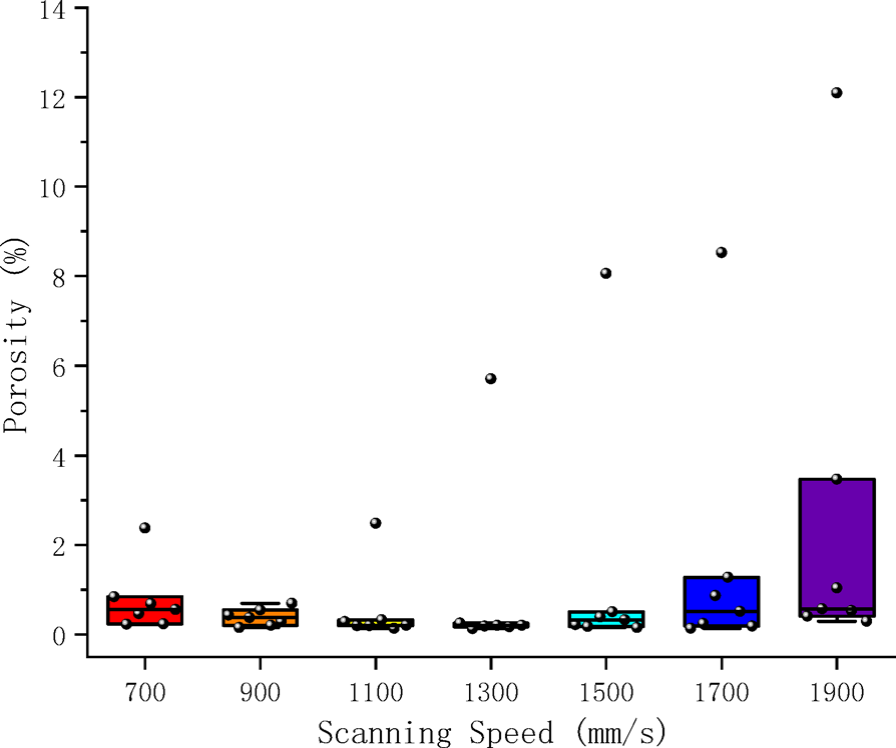
Scatter box plot of porosity and scanning speed.

**Fig. 10:**
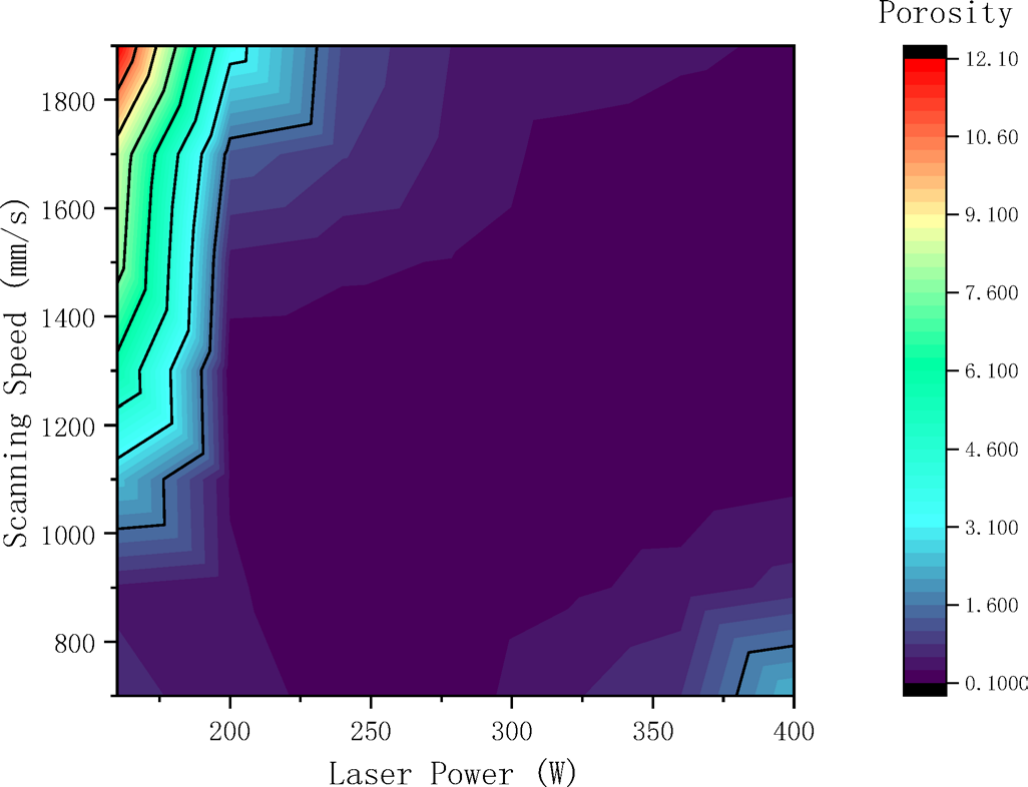
Two-dimensional contour plot of porosity and process parameters.

To explore the correlation between process parameters and pore defects, the proposed model was employed to analyze micrographs of all samples, quantifying the porosity characteristics for each specimen. Figures 8 and 9 displays scatter box plots illustrating the distribution relationship between porosity and individual process parameters, indicating that porosity is significantly influenced by process parameters. Additionally, Fig. 10, 11 presents a two-dimensional contour plot and three-dimensional analysis diagram of porosity versus laser power and scanning speed. The laser power range spans from 160 to 400 W, while the scanning speed range extends from 700 to 1900 mm/s. Figure 12 presents a scatter plot illustrating the distribution relationship between porosity and energy density.

**Fig. 11:**
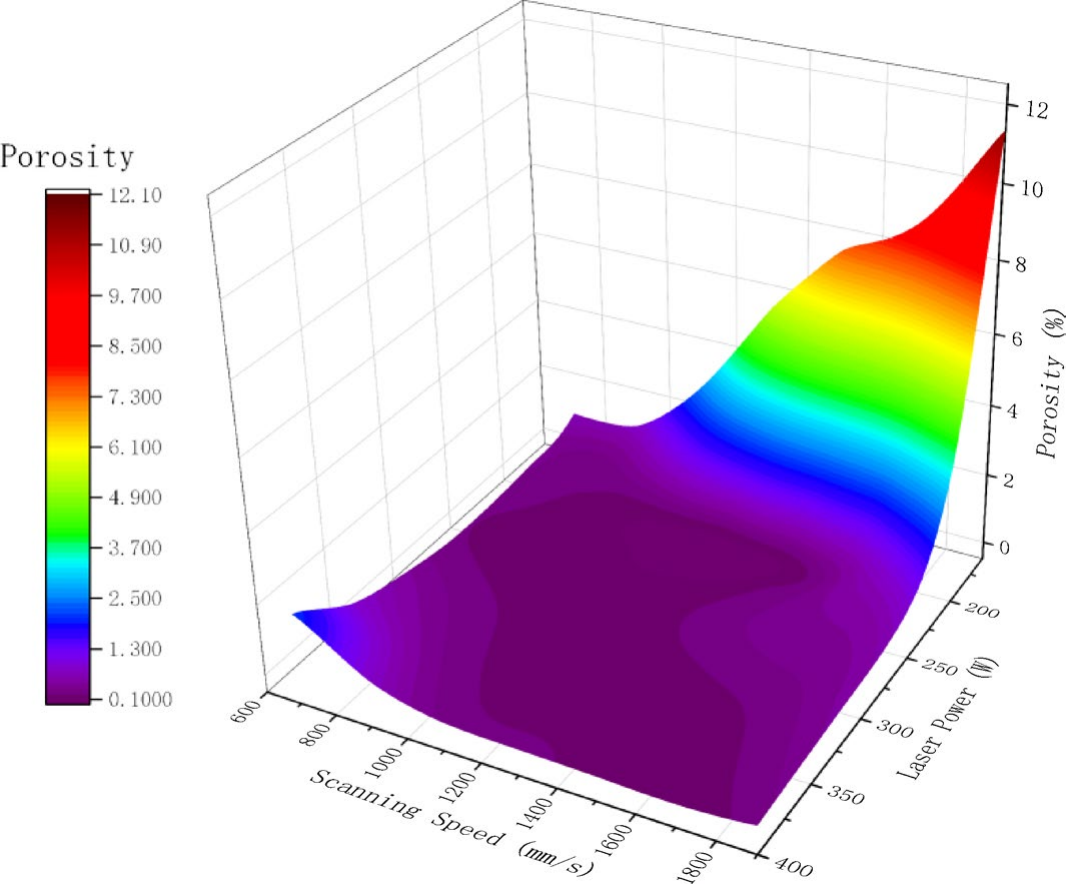
Three-dimensional analysis diagram of porosity and process parameters.

**Fig. 12:**
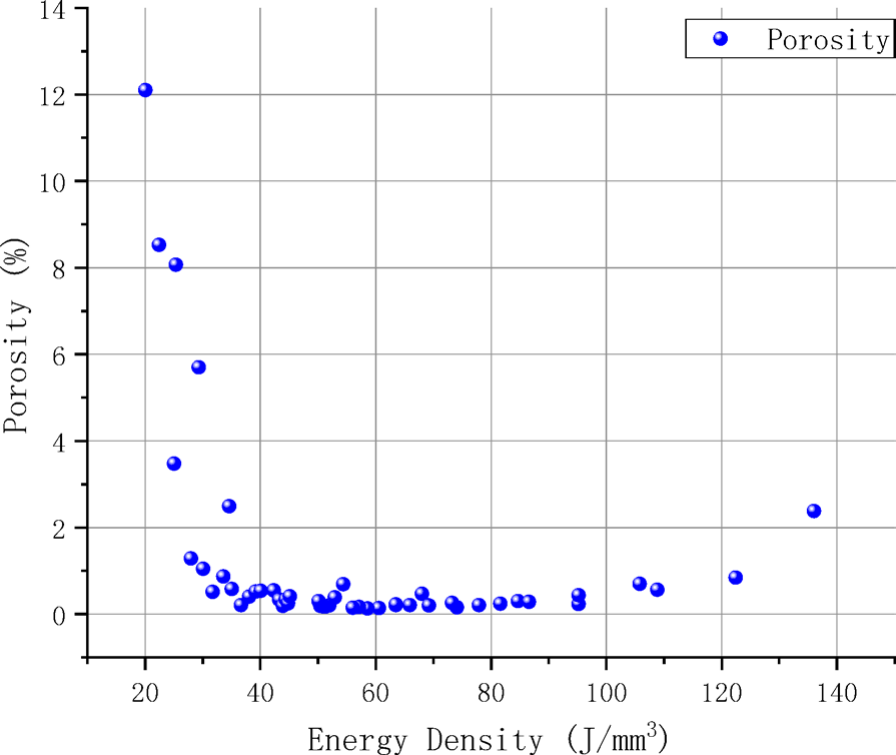
Scatter plot of porosity and energy density.

Laser power is a critical energy input parameter on SLM manufacturing, directly influencing the thermodynamic behavior of the melt pool and thereby altering the tendency for porosity formation.

As revealed in Fig. 7, the increase in laser power delivers enhanced energy input, promoting more thorough melting of the powder particles and prolonging the existence time of the molten pool. This provides additional time for gases generated during manufacturing to diffuse and escape, significantly reducing the probability of porosity caused by incomplete melting and insufficient gas venting. Simultaneously, the increased power amplifies the intensity of Marangoni convection within the molten pool, aiding in pushing gases toward the pool surface for complete escape.

However, the positive effects of laser power increase exhibit distinct nonlinear characteristics. As shown in Fig. 8, when laser power exceeds a certain threshold (approximately 360W), further increases may instead cause excessive oscillation of the molten pool, leading to increased porosity. Notably, the porosity becomes more sensitive to laser power variations in the low-power range, such as below 200W. Laser power increases in the 160-200W range significantly reduce porosity (by approximately 7%) overall, whereas the reduction weakens to 1% in the 200-360W range. This characteristic explains why the rate of porosity reduction gradually diminishes within the positive influence range of increasing laser power. While appropriately raising the laser power can significantly mitigate pore defects, the improvement effect tends to weaken as the power level continues to increase.

Scanning speed influences porosity formation dynamics by regulating the temporal scale of energy input. Lower scanning speeds provide longer interaction time between the energy and material, prolonging the molten pool’s duration to enhance gas diffusion. This reduces melt viscosity and improves gas escape conditions. When scanning speeds increase beyond a certain threshold, the abrupt reduction in energy input time leads to numerous adverse effects such as reduced molten pool lifetime and rapid solidification of the melt, which hinders the upward movement of gas bubbles. Consequently, the porosity increases. As shown in Fig. 9, porosity remains relatively low at lower scanning speeds, but begins to rise rapidly once the speed exceeds a critical value.

In addition, the impact of scanning speed varies across different power levels. Under 160W laser power conditions, changes in scanning speed exert a more pronounced effect on porosity percentage. For example, when the speed increases from 1100 mm/s to 1900 mm/s, the porosity rises by 10%. Conversely, under 280W laser power conditions, the same speed change only increases porosity by approximately 0.4%. This indicates that higher power levels can partially offset the adverse effects of elevated scanning speeds.

Analysis of the three-dimensional process window (Figs. 10, 11) revealed a distinct nonlinear coupling effect between laser power and scanning speed, which influences porosity formation through energy density. Figure 11 demonstrates that low power, high scanning speed processes tend to exhibit lack of fusion defects, while high power, low scanning speed conditions are prone to keyhole formation. As revealed Fig. 12, a stable low porosity (approximately less than 1%) is achievable within the energy density range of 60–90 J/mm^3^. When the energy density exceeds 100 J/mm^3^, porosity begins to increase, though at a relatively modest rate. Conversely, porosity remains elevated when the energy density falls below 40 J/mm^3^, primarily due to insufficient input energy causing instability in the molten pool and resulting in numerous defects. A two-variable polynomial fitting model based on laser power and scanning speed is established using Chebyshev series Ln X, Y binomial polynomials. Normalized data for laser power and scanning speed were utilized, yielding the following specific model:

4 $$Z = \mathop \sum \limits_{m = 0}^{6} \mathop \sum \limits_{n = 0}^{6} a_{mn} T_{m} \left( {ln\left( x \right)} \right)T_{n} \left( y \right)$$

$$T_{m} \left( {ln\left( x \right)} \right) = cos\left( {m*acos\left( {ln\left( x \right)} \right)} \right)$$

$$T_{n} \left( y \right) = cos\left( {n*acos\left( y \right)} \right)$$

In Eq. (4), *x* = *ln*(*x*) represents laser power normalized within the range of -1 to 1. *y* represents scanning speed normalized within the range of -1 to 1. *z* serves as porosity. *a*_*mn*_ denotes the fitted coefficient. *m* and *n* are integers ranging from 0 to 6, which means the model is a sixth-order binomial polynomial. Table 5 provides the fitted coefficient matrix.

**Table 5:** Fitted coefficient matrix.

a_mn_		m						
		0	1	2	3	4	5	6
n	0	1.34339	− 1.63507	1.21365	− 0.57853	0.35102	− 0.14641	0.07781
	1	1.19112	− 2.39564	1.13576	− 0.76657	0.24563	− 0.15499	0
	2	0.40745	− 0.14269	0.09808	0.21711	− 0.05943	0	0
	3	− 0.07628	0.00736	− 0.20202	0.08566	0	0	0
	4	0.18761	− 0.23304	0.22316	0	0	0	0
	5	0.07351	− 0.12553	0	0	0	0	0
	6	0.01478	0	0	0	0	0	0

The model achieved a coefficient of determination R2 = 0.992, indicating its effective ability to explain the nonlinear relationship between process parameters and porosity. While the model exhibits slight fluctuations in characterizing patterns within the porosity range, it performs well in the high porosity range, demonstrating overall stability.

### Limitation analysis

This study proposes an additive manufacturing porosity analysis method combining instance segmentation and polynomial fitting, demonstrating its technical feasibility under laboratory-controlled conditions. However, practical application in industrial settings presents notable constraints. First, surface treatment techniques like grinding and polishing are often inapplicable in online/nearline inspection; second, optical microscopy systems face inherent limitations in field-of-view and depth-of-field, with imaging quality varying across different part surfaces.

Regarding material applicability, the proposed method segments defects based on morphological features rather than material-specific properties, theoretically enabling cross-material generalization. Nevertheless, surface conditions post-processing inevitably vary due to material property differences and process parameter variations, which may degrade segmentation performance—particularly when applying unprocessed industrial field images directly. Consequently, the model requires additional training with a dataset of newly supplemented images to adapt to emerging segmentation requirements. Moreover, while manual annotation remains a primary labeling approach, its consistency is challenged by operator expertise variability and subjective judgment biases.

To address these limitations, future research will focus on three directions. Robustness enhancement: Improving interference resistance against unprocessed surfaces and material-induced image variations to strengthen industrial applicability; Adaptive strategies: Developing transfer learning frameworks to reduce retraining demands for new materials; Advanced labeling: Establishing standardized annotation protocols through multi-dimensional data fusion, integrating three-dimensional pore data from XCT to generate hybrid labels that enrich dataset diversity and enhance model precision. The follow-up will systematically address three critical challenges—industrial adaptability, cross-material generalization, and dataset labeling consistency—to bridge the gap between laboratory validation and production deployment.

## Conclusion

To address the challenge of detection and analysis of pore defects for SLM fabricated parts, a collaborative analysis method integrating instance segmentation and polynomial fitting has been designed. An enhanced instance segmentation model is proposed. By establishing a novel adaptive curve learning rate adjustment strategy, a feature extraction module, and a lightweight feature fusion network, the model’s capability for feature extraction from complex input data and its learning iteration strategy are improved. While maintaining relatively low model complexity, this approach significantly enhances the segmentation accuracy of pore defects and enables automatic calculation of the pixel area proportion of porosity in images. A novel SLM pore defect image dataset is constructed through image acquisition and manual annotation. Comparative validation experiments conducted on this dataset demonstrated the proposed model’s robust performance (mAP reaching 85.6%), providing crucial data support for related research. Utilizing pore defect data obtained from the segmentation model, we conducted an in-depth investigation into the influence rule of laser power and scanning speed on pore defects. A mapping model linking process parameters to porosity density is established using a sixth-order Chebyshev binomial polynomial, achieving a coefficient of determination R2 of 0.992. The research findings demonstrate that this method enables precise identification and quantitative analysis of pore defects, offering a reliable new approach for analyzing SLM process parameters. It holds significant academic value and promising engineering applications, though several areas warrant further exploration.

Future research can be conducted in the following areas: expanding the dataset to include more pore defect samples under various process conditions; further refining the model to adapt to more complex and diverse image processing scenarios; developing adaptive process optimization methods by integrating optimization algorithms; and establishing a closed-loop control process for pore defect analysis and process optimization. These future research efforts will enhance the reliability and universality of this method in industrial applications, providing robust technical support for quality control in metal additive manufacturing. The scale of dataset and hyperparameter configurations play critical roles in model training. To address this, we will investigate synthetic data generation methodologies based on physics-informed or diffusion models, while expanding the existing dataset through benchmark data collection across diverse material types. Furthermore, we will conduct systematic hyperparameter optimization tailored to the instance segmentation task for additive manufacturing porosity defects, integrating advanced training strategies to enhance model robustness and adaptability in industrial deployment scenarios.

## Data Availability

Data available on request from the authors.
